# *De Novo* Genome Assembly of *Auanema Melissensis*, a Trioecious Free-Living Nematode

**DOI:** 10.2478/jofnem-2022-0059

**Published:** 2023-02-07

**Authors:** Sophie Tandonnet, Maairah Haq, Anisa Turner, Theresa Grana, Panagiota Paganopoulou, Sally Adams, Sandhya Dhawan, Natsumi Kanzaki, Isabelle Nuez, Marie-Anne Félix, André Pires-daSilva

**Affiliations:** 1 School of Life Sciences, University of Warwick, Coventry, CV4 7AL, UK; 2 Department of Biological Sciences, University of Mary Washington, Fredericksburg, VA 22401 UK; 3 Kansai Research Center, Forestry and Forest Products Research Institute, Fushimi, Kyoto 612-0855, Japan; 4 Institut Jacques Monod, CNRS UMR7592, Université Paris-Diderot, 75013 Paris, France

**Keywords:** annotation, assembly, *Auanema melissensis*, genomics, morphology, nematode, taxonomy, trioecious

## Abstract

Nematodes of the genus *Auanema* are interesting models for studying sex determination mechanisms because their populations consist of three sexual morphs (males, females, and hermaphrodites) and produce skewed sex ratios. Here, we introduce a new undescribed species of this genus, *Auanema melissensis* n. sp., together with its draft nuclear genome. This species is also trioecious and does not cross with the other described species *A. rhodensis* or *A. freiburgensis*. Similar to *A. freiburgensis*, *A. melissensis*’ maternal environment influences the hermaphrodite versus female sex determination of the offspring. The genome of *A. melissensis* is ~60 Mb, containing 11,040 protein-coding genes and 8.07% of repeat sequences. Using the estimated ancestral chromosomal gene content (Nigon elements), it was possible to identify putative X chromosome scaffolds.

Nematodes of the genus *Auanema* are free-living species that have an unusual trioecious mating system, e.g., their populations are composed of self-reproducing hermaphrodites, outcrossing females, and males (Félix, 2004; Chaudhuri et al., 2011a; Kanzaki et al., 2017; Winter et al., 2017). In laboratory cultures, XO males are in lower proportions (typically 5–15%) than XX non-males (females and hermaphrodites) (Félix, 2004; Chaudhuri et al., 2011a, 2015; Shakes et al., 2011; Robles et al., 2021). The dynamics of this unusual mating system is poorly understood (Anderson et al., 2020; Rupp, 2020). In the species described so far, *Auanema rhodensis* (aka *Rhabditis* sp. SB347) and *Auanema freiburgensis* (aka *Rhabditis* sp. SB372), the passage through a dauer larval stage is obligatory for hermaphrodite development (Félix, 2004; Chaudhuri et al., 2011a; Zuco et al., 2018; Robles et al., 2021). The mechanisms linking the dauer pathway to the sex determination pathway are still not known.

*Auanema* species display atypical segregation and inheritance patterns of the X chromosome, which is gametogenesis- and sexual morph-dependent (Shen and Ellis, 2018; Tandonnet et al., 2018). These singularities lead to skewed sex ratios according to the reproduction mode (crossings and selfing) (Tandonnet et al., 2018). In *A. rhodensis*, the three sexual morphs are produced by selfing hermaphrodites and crossing females, independently of the environmental conditions (Chaudhuri et al., 2015). In *A. freiburgensis*, however, mothers exposed to crowding cues derived from high population densities produce mostly hermaphrodite offspring. In the absence of those crowding cues, mothers produce predominantly females and a few males (Zuco et al., 2018; Robles et al., 2021).

The genomes of *A. rhodensis* and *A. freiburgensis* are relatively small (~60 Mb) and organized into seven chromosomes (Tandonnet et al., 2019; Al-Yazeedi, pers. comm.). This number of chromosomes differs from the usual six, which is the karyotype of most other nematodes in the same clade (Clade V) (Gonzalez de la Rosa et al., 2021). By tracking the ancestral linkage groups in nematode evolution, the so-called Nigon elements, it is known that *Auanema* chromosomes have undergone more fission and fusion events than other clade V nematodes (Tandonnet et al., 2019; Gonzalez de la Rosa et al., 2021). The reasons for this evolutionary pattern are unknown.

In this study, we report a new species of the genus *Auanema*, which we named *Auanema melissensis*, along with its draft genome assembly and associated annotation. We also describe the morphological and biological characteristics of this nematode and compare them to those of *A. rhodensis* and *A. freiburgensis*.

## Materials and Methods

### Nematode culture

*Auanema melissensis* (aka, *Auanema* sp. JU1783) was maintained under the standard culture conditions of *Caenorhabditis elegans* (Chaudhuri et al., 2011b) at 20°C. Plates were seeded with the streptomycin-resistant *Escherichia coli* strain OP50-1. Microbial contamination was prevented by adding 50 mg/ml of streptomycin and 10 mg/ml of nystatin to the nematode growth medium (NGM).

### Determination of Trioecy

To determine if *A. melissensis* was trioecious, we randomly isolated eggs from the culture plates. The eggs were individually placed into 48-well plates seeded with *Escherichia coli* OP50-1 and allowed to develop to adulthood in isolation. The sexual morph was determined by morphological characters and the ability or not to reproduce on their own. Hermaphrodites laid eggs in the absence of a mating partner, whereas females could only reproduce if paired with a male. Males were distinguished by their blunt tails.

### The sexual fate of dauer larvae

Dauers of other *Auanema* species invariably develop into self-reproducing hermaphrodites (Félix, 2004; Chaudhuri et al., 2011a; Zuco et al., 2018; Robles et al., 2021). To determine if this was also the case in *A. melissensis*, we isolated dauers onto individual plates seeded with *E. coli* OP50-1 and determined their sexual morph in adulthood, as described above.

### Effect of crowding cue on the female/ hermaphrodite ratio

To test if the crowding conditions have an effect on the proportion of each sexual morph produced, we counted the number of male, female and hermaphrodite progeny produced by hermaphrodite mothers placed in the presence and absence of crowding cues. The crowding cues were prepared by first washing a Petri dish (ϕ = 60 mm) containing a crowded culture of nematodes with 1 ml of M9 buffer. The resuspended nematode culture was placed in a 50 ml tube and left on a thermomixer for 16–24 hr at 20°C. The liquid culture was then transferred to a 1.5 ml microcentrifuge tube and centrifuged for 45 min, at 15,000 rpm. Once clear from nematodes, the supernatant with the crowding cues was placed in a clean 1.5 ml tube and used immediately.

For the treatment conditions, we used Petri dishes (ϕ = 60 mm) with NGM that were seeded with 50 ml of OP50-1 and supplemented with either 100 ml of supernatant with crowding cues or with M9 buffer (control). The crowding cue or M9 buffer was added directly to the OP50-1 lawn in two installments of 50 ml, letting the liquid dry after each installment.

Dauers (fated to become hermaphrodites) were isolated separately on either the “crowding conditions” or the “control conditions” plates and allowed to develop into adulthood at 20°C. Hermaphrodite mothers were moved to a new plate (under the same conditions) each day and egg collection was carried out >3 d (Fig. S1 in Supplementary Materials). Eggs were placed individually on standard (non-treated) plates and the sexual morph was identified as described above.

**Figure S1:**
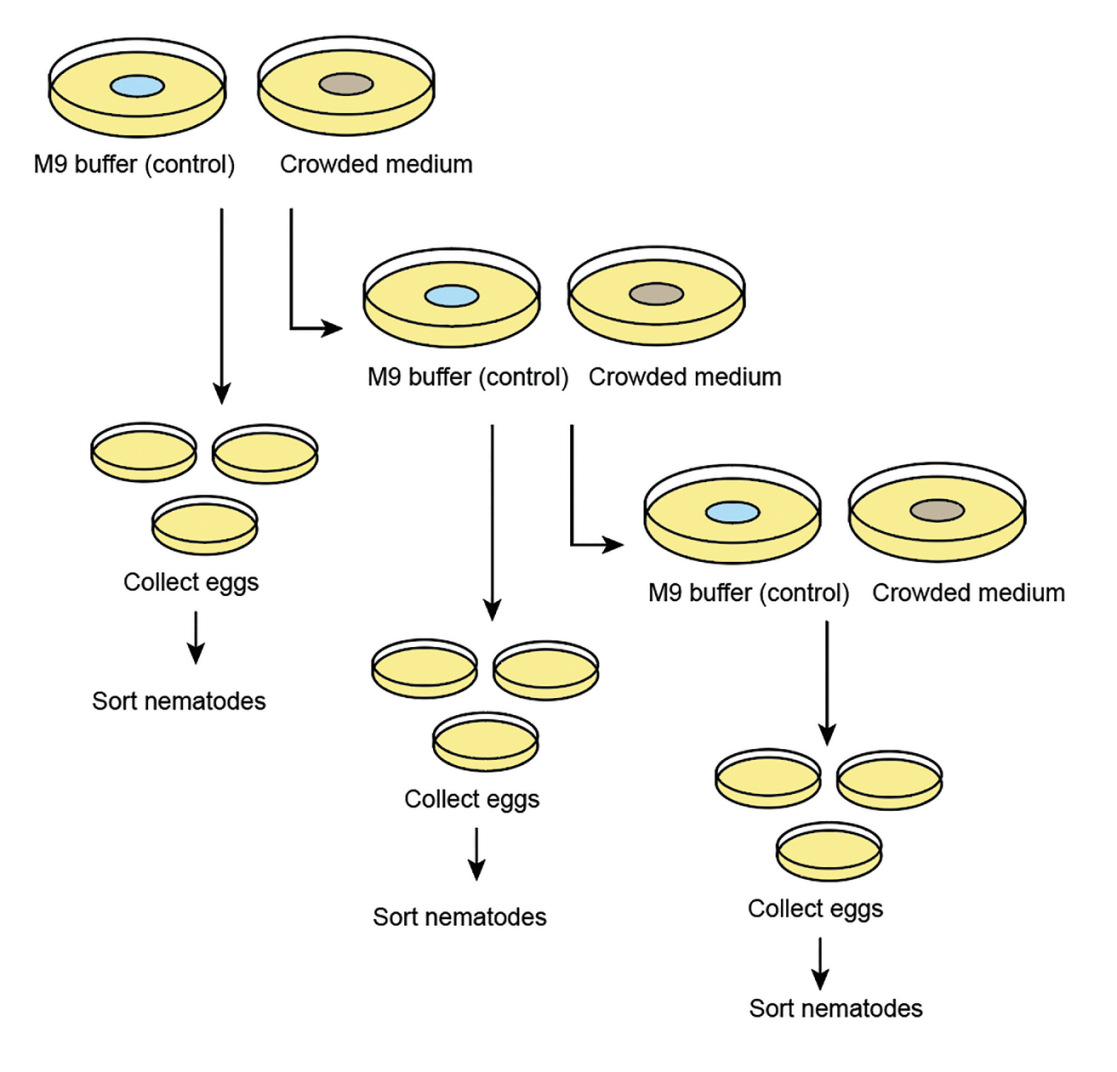
Diagram of the experimental design to test if the crowding cues experienced by hermaphrodite mothers change the sex ratios of the F1 generation. Dauer larvae were isolated onto five 6 cm plates under each condition (control and crowded) and left to mature into a hermaphrodite. F1 eggs were then collected and placed individually onto non-treated 3.5 cm plates. Egg collection occurred >3 d and hermaphrodite mothers were moved to new plates (of their original condition) each day. Eggs were allowed to develop until adulthood and sexed.

We built a generalized linear model (GLM) in R using the “glm” function to test if the proportion of females and hermaphrodites depends on the experimental conditions. The data were fitted to a binomial distribution.

### Microscopy and whole-body measurements

Pictures of ten males, hermaphrodites, and females were taken on day 1 of adulthood with a Zeiss Axio Zoom V16 microscope, using a microscopy camera Axiocam and processed with the Zeiss ZEN2 software. Pictures were stored and edited in TIFF file formats. All pictures were taken at 100× magnification.

Measurements of whole-body adults of each sexual morph were taken using the ImageJ “Measure” function. Nematode length was measured, head to tail, and the scale was defined using image scale bars and the ImageJ “Set Scale” feature.

### Time course

A time course following ten females and ten hermaphrodites over the first 3 d of adulthood was also conducted using the same microscope settings stated above. Measurements of whole-body adults were taken in two parts: from the tip of the head to the anus and from the anus to the tip of the tail. This avoided measurement errors in pictures where the worm had an extremely curled tail. We analyzed the data by performing a two-way repeated measures ANOVA to evaluate simultaneously the effect of the sexual morph and the age on the total body length variable. A repeated measures ANOVA was necessary as the same individuals were measured for three consecutive days. The normality of the data was assessed by performing Shapiro–Wilk tests. We performed pairwise comparisons (*t*-tests) to further analyze the differences between age and sex.

### Male tail imaging

Typological characters of the male tail, which are considered to represent species-specific characters, were observed and micrographed using a light microscope Eclipse Ni (Nikon) facilitated with DIC optics and a digital camera MC170 HD (Leica) attached to the microscope. Dauer larvae were individually cultured on small (ϕ = 40 mm) Petri dishes and allowed to develop into self-reproducing hermaphrodites. Newly emerged F1 males were picked up using a stainless steel insect pin (Insect pin #00, Shiga Kontyu), and observed and micrographed using the silicon grease method (Kanzaki, 2013). Micrographs were edited with PhotoShop Elements 2021 (Adobe) to construct the figure plate.

### Preparation of fixed museum specimens

The type material of each sexual morph was prepared according to the Natural History Museum of London specifications. Ten individuals of each sexual morph were placed in a microcentrifuge tube with 200 ml of 80% ethanol.

## DNA and RNA extraction

A large mixed nematode population of *A. melissensis* cultured on NGM agarose plates was used to extract the genomic DNA. The nematode handling and DNA extraction were based on the genomic DNA preparation protocol from Dudley and Goldstein (2005) except for pelleting the DNA by centrifugation at 14,000 rpm for 15–30 min instead of winding out the DNA precipitate. After removing the supernatant, an ethanol precipitation step was performed (i.e., the DNA pellet was washed using cold 70% ethanol and centrifuged before discarding the supernatant).

For RNA extraction, we also used a mixed population of nematodes reared on NGM agar plates. Plates were washed using M9 and worms were pipetted into a 15 ml conical tube. The tube was centrifuged for 15 min to gather the nematodes in a “pellet” at the bottom of the tube. The “supernatant” was discarded, and the nematodes were transferred into 1.5 ml tubes. The tubes were placed on dry ice until RNA extraction. For RNA extraction, the nematodes were resuspended in 1 ml of lysis buffer and shredded by sonication until the solution was bubbly and homogeneous (five cycles). Subsequently, we used the RNAeasy Mini kit, following the manufacturer’s protocol.

### Illumina sequencing and preprocessing

An Illumina whole genome sequencing (WGS) pair-end library of insert-size 200 bp and three mate-pair Illumina WGS libraries of insert-sizes 3 kb, 5 kb, and 8 kb were sequenced using a HiSeq2000 sequencer (Table S1 in Supplementary Materials). Raw reads were preprocessed using Skewer, version 0.2.2 (Jiang et al., 2014) to remove poor-quality sequence regions (<20) and reads smaller than 51 bases (Table S2 in Supplementary Materials). A preliminary genome was assembled to estimate the libraries’ insert sizes and assess contamination. We used Blobtools, version 1.1.1 (Laetsch and Blaxter, 2017) to visualize and assess contamination levels (Fig. S2 in Supplementary Materials). Our libraries contained little contaminants (<2% of the trimmed reads), which predominantly corresponded to *E. coli*. We assumed it was the strain OP50-1 as this strain was used as the food source of the nematode culture. The reads mapping to the genome of OP50-1 (GCA_000176815.1, ASM17681v1) were removed and not used for the final genome assembly (Table S2 in Supplementary Materials). Reads were error-corrected using Fiona, version 0.2.10 (Schulz et al., 2014) using 60 Mb as an estimated genome size.

**Figure S2:**
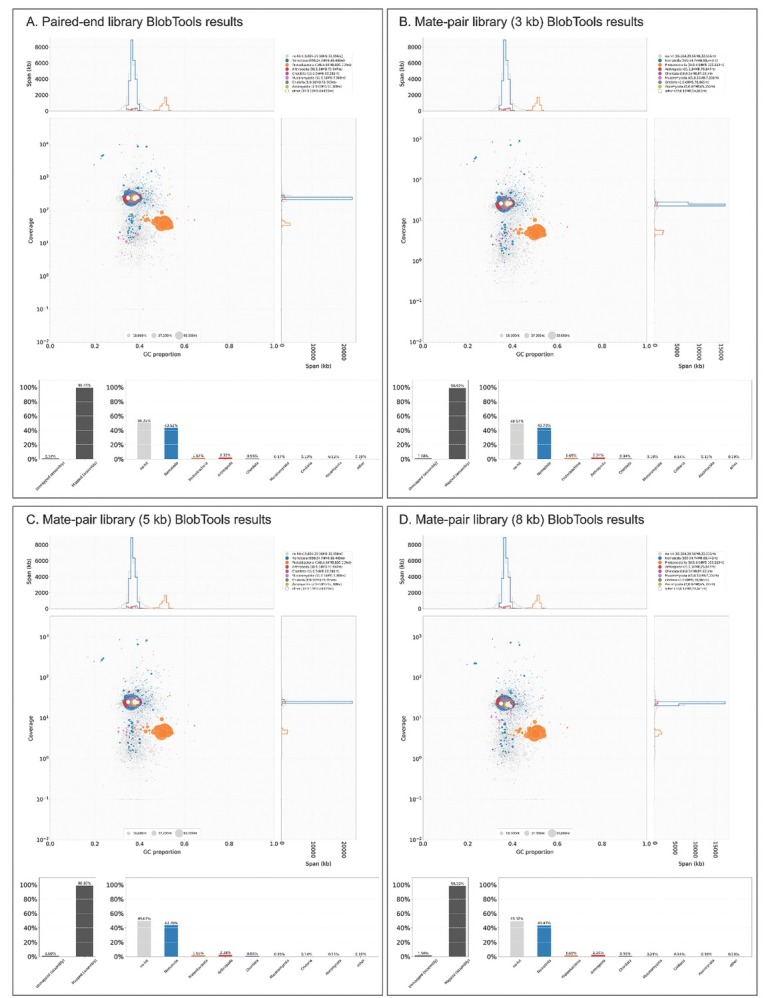
Visualization of the contamination present in the four genomic libraries using BlobTools. The libraries were generally free from contaminants except for a few (<2%) proteobacteria most likely corresponding to the nematode food source (*E. coli* strain OP50-1).

**Table S1:** Genomic and transcriptomic raw data used in this study.

Library (insert size)	Number of raw pairs	GC content (%)	Run accession number (experiment acc)
Pair-end DNA library (200 bp)	68,587,064	37	ERR9709439 (ERX9258761)
Mate-pair DNA library (3 kb)	16,195,093	37	ERR9709942 (ERX9259264)
Mate-pair DNA library (5 kb)	11,655,487	37	ERR9710904 (ERX9260226)
Mate-pair DNA library (8 kb)	10,769,133	37	ERR9709954 (ERX9259276)
RNA-seq library of mixed stages	22,509,195	44	ERR9712246 (ERX9261548)

**Table S2:** Preprocessing of the genomic libraries.

Library (insert size)	Number of raw pairs	Number of pairs after trimming (quality and size)*	Number of pairs after contamination removal
Pair-end DNA library (200 bp)	68,587,064	68,109,105 (99.30%)	67,271,297
Mate-pair DNA library (3 kb)	16,195,093	9,920,448 (61.26%)	9,766,526
Mate-pair DNA library (5 kb)	11,655,487	7,484,279 (64.21%)	7,372,147
Mate-pair DNA library (8 kb)	10,769,133	7,125,674 (66.17%)	7,017,505

*Reads were trimmed for quality (> 20) and length (> 51 bases).

An Illumina paired-end RNA-seq library from a mixed population was sequenced using an Illumina NovaSeq 6000 sequencer (Table S1 in Supplementary Materials). Mature RNA was selected using an oligo-dT method. Raw reads were preprocessed using Skewer, version 0.2.2 (Jiang et al., 2014) to remove poor-quality sequences.

## Genome assembly

The final *de novo* genome assembly was performed with MEGAHIT, version 1.2.6 (Li et al., 2015, 2016) followed by SOAPdenovo, version 2.04-r241 (Luo et al., 2012) with a k-mer length of 35, using the paired-end library for contig assembly and the mate-pair libraries for scaffolding, as this resulted in the best assembly strategy tested. Gaps were closed using SOAPdenovo’s GapCloser. Genome completeness was assessed using BUSCO, version 5.2.2 using the nematoda_obd10 database (Simão et al., 2015).

### Genome Annotation of repeats and protein-coding genes

Repeats were identified and soft-masked using a combination of programs: TransposonPSI (http:// transposonpsi.sourceforge.net/), LTR_harvest (Ellinghaus et al., 2008) and LTR_Digest (Steinbiss et al., 2009), LTR_finder (version 1.0.7) (Xu and Wang, 2007), RepeatModeler (version 2.0.1) (Flynn et al., 2020) (https://www.repeatmasker.org/RepeatModeler/) and RepeatMasker (version 4.0.9-p2) (https://www.repeatmasker.org/).

The masked genome was then annotated using BRAKER2 version 2.1.6 (Brůna et al., 2021), which uses evidence-based data in conjunction with the *ab initio* predictors GeneMark (Brůna et al., 2020) and Augustus version 3.4.0 (Stanke et al., 2006). The first run of BRAKER2 was performed using transcriptomic data (RNA-seq reads and de-novo assembled transcriptome). The RNA-seq data generated in this study were pre-processed using Skewer (version 0.2.2) (Jiang et al., 2014) and the trimmed reads were used directly as gene hints by BRAKER2. We also assembled a *de novo* transcriptome from the trimmed RNA-seq reads using Trinity (version 2.10.0) (Grabherr et al., 2011). The second run of BRAKER2 used the curated protein database “metazoa_odb10” database from orthoDB (Grabherr et al., 2011; Kriventseva et al., 2019) as the evidence-based data. The results of both runs were merged using TSEBRA (Gabriel et al., 2021).

### Phylogenetic position of *A. melissensis*

The phylogenetic position of *A. melissensis* was determined in relation to other Clade V nematode species: *C. elegans* (WBcel235, GCF_000002985.6), *Oscheius tipulae* (GCA_013425905.1), *Pristionchus pacificus* (el paco v4, GCA_000180635.4), *A. rhodensis* (GCA_947366455), and *A. freiburgensis* (Talal Al Yazeedi, pers. comm.), using BUSCO single-copy orthologs determined through the Busco Phylogenomics script of Jamie McGowan (https://github.com/jamiemcg/BUSCO_phylogenomics). Briefly, after running BUSCO version 5.2.2 (Simão et al., 2015) on all genomes (Table S3 in Supplementary Materials), single-copy BUSCOs found in at least four species were used to construct alignments using MUSCLE (Edgar, 2004). The alignments were trimmed with TrimAl (Capella-Gutiérrez et al., 2009) and used to construct a phylogenetic tree with either a supertree or a supermatrix approach. For the supertree approach, maximum likelihood trees were constructed for each BUSCO family using the IQ-Tree (Minh et al., 2020). The resulting BUSCO phylogenies were concatenated with ASTRAL, version 5.7.8 (Zhang et al., 2018) to generate the final supertree species’ phylogenetic relationships. For the supermatrix approach, we constructed the phylogenetic tree using Fasttree2 (option “-pseudo,” version 2.1.11) on the concatenated trimmed alignments.

**Table S3:** BUSCO scores of the genomes used to construct the phylogeny.

	Complete	Complete and single copy	Complete and duplicated	Fragmented	Missing
*A. melissensis* (this study, GCA_943334845.1)	2,784 (88.9%)	2,771 (88.5%)	13 (0.4%)	45 (1.4%)	302 (9.7%)
*A. freiburgensis* (Talal Al Yazeedi, pers. comm.)	2,789 (89.1%)	2,756 (88.0%)	33 (1.1%)	48 (1.5%)	294 (9.4%)
*A. rhodensis* (GCA_947366455)	2,812 (89.8%)	2,796 (89.3%)	16 (0.5%)	39 (1.2%)	280 (9.0%)
*O. tipulae* (GCA_013425905.1)	2,843 (90.8%)	2,786 (89.0%)	57 (1.8%)	47 (1.5%)	241 (7.7%)
*C. elegans* (GCF_000002985.6)	3,113 (99.4%)	3,096 (98.9%)	17 (0.5%)	3 (0.1%)	15 (0.5%)
*P. pacificus* (GCA_000180635.4)	2,587 (82.6%)	2,537 (81.0%)	50 (1.6%)	38 (1.2%)	506 (16.2%)

The “nematoda_odb10” (containing 3,131 BUSCOs) database was used.

## Results

### Species description

**Figure 1:**
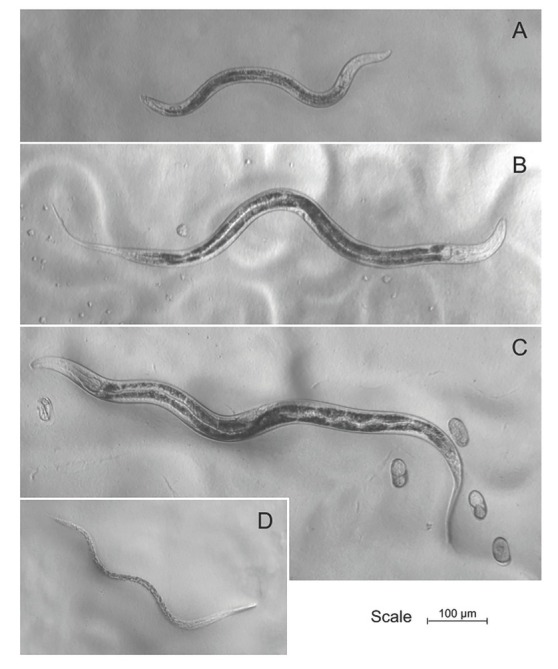
Pictures of a (A) male, (B) female, and (C) hermaphrodite of *Auanema melissensis* on day 1 of adulthood (100× magnification). A dauer larva (D), fated to develop as a hermaphrodite, is depicted at a magnification of 112×.

**Figure 2:**
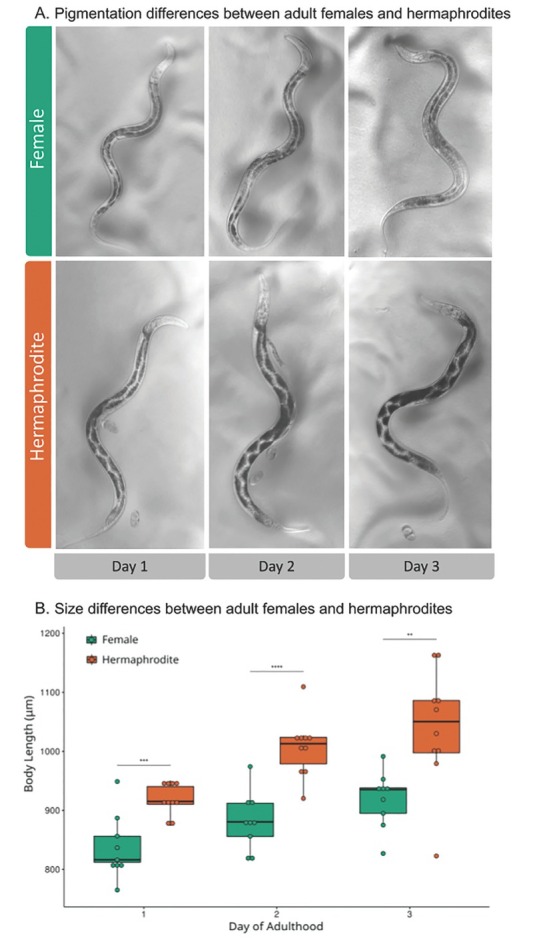
Differences between females and hermaphrodites during the first three days of adulthood. (A) Representative example of the size and pigmentation differences between *A. melissensis* females and hermaphrodites over the first 3 d of adulthood (100× magnification). The scale bar is the same for all images. (B) Hermaphrodites’ body length is longer than that of females during the first three days of adulthood (repeated measures ANOVA F(1, 8) = 26.580, *P* < 0.05). As the worms aged, they also became longer (repeated measures ANOVA F(2, 16) = 19.151, *P* < 0.05).

**Figure 3:**
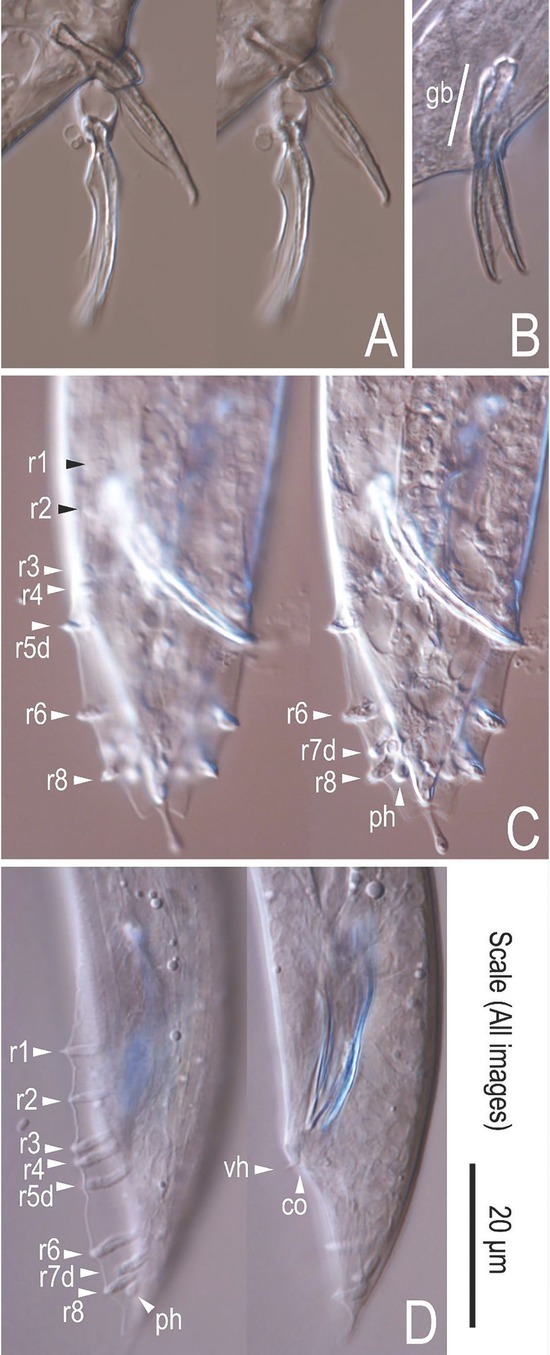
Male tail characters of *A. melissensis*. Micrographs (A) and (B) show the spicules. Male tail in ventral (C) and lateral (D) orientation. “ph” = Phasmid, “CO” = cloaca, “gb” = gubernaculum, “vh” = ventral hook. Genital papillae/rays are designated by arrowheads and named r1-r8.

*Auanema melissensis* n. sp. (Figs. 1, 2A, and 3) (=*Auanema* sp. JU1783) typologically fits the generic character of *Auanema* (Kanzaki et al., 2017). Therefore, only body size and some diagnostic characteristics are described here. Whole body length differed between the sexual morphs at day 1 of adulthood: males were small (437.2 ± 18.4 mm, *n* = 10) and females (854.0 ± 77.0 mm, *n* = 10) were found to be smaller than hermaphrodites (963.1 ± 64.0 mm, *n* = 10) (Wilcoxon Rank-Sum exact test, W = 11; *P*-value = 0.002089; Table S4 and Fig. S3 in Supplementary Materials). A time course following 10 females and 10 hermaphrodites >3 d revealed that the size difference between the non-male sexual morphs was maintained later in adulthood (repeated measures ANOVA F(1, 8) = 26.580, *P* < 0.05, Fig. 2).

**Figure S3:**
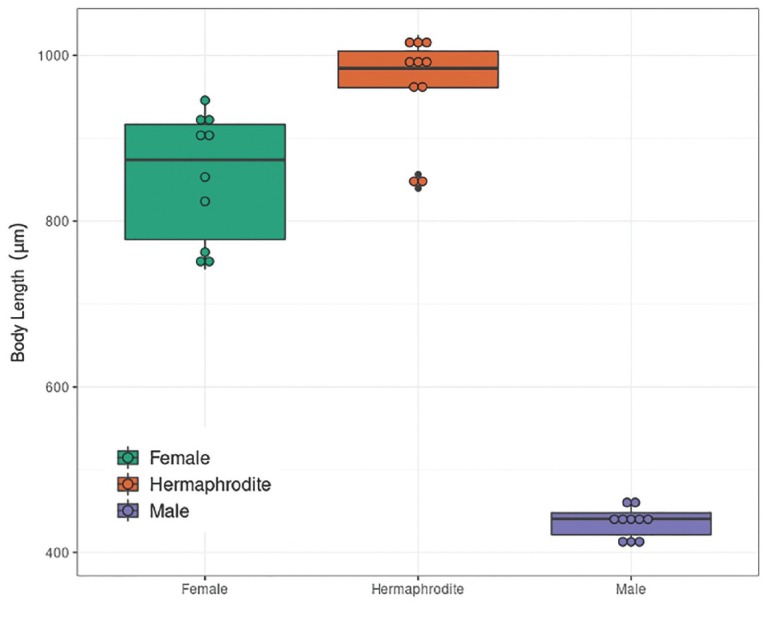
Whole body length of males (*n* = 10), females (*n* = 10) and hermaphrodites (*n* = 10).

**Table S4:** Whole body measurements of ten males, females and hermaphrodites.

ID	Sexual morph	Length (μm)
MM1	Male	408.007
MM2	Male	417.763
MM3	Male	459.784
MM4	Male	416.338
MM5	Male	448.795
MM6	Male	440.899
MM7	Male	461.569
MM8	Male	446.739
MM9	Male	431.713
MM10	Male	440.548
FM1	Female	823.88
FM2	Female	762.546
FM3	Female	761.116
FM4	Female	853.252
FM5	Female	945.703
FM6	Female	918.242
FM7	Female	894.754
FM8	Female	912.291
FM9	Female	741.757
FM11	Female	926.158
HM1	Hermaphrodite	1,006.355
HM2	Hermaphrodite	960.354
HM3	Hermaphrodite	1,001.369
HM4	Hermaphrodite	1,024.498
HM5	Hermaphrodite	856.356
HM6	Hermaphrodite	1,010.322
HM7	Hermaphrodite	839.704
HM8	Hermaphrodite	986.148
HM10	Hermaphrodite	982.561
HM11	Hermaphrodite	963.425

Measurements were taken from the tip of the head to the tip of the tail.

We also observed a difference in pigmentation between females and hermaphrodites, which accentuated during the time course: hermaphrodites seemed to accumulate pigments in the gut whereas females may effectively lose them (Fig. 2B). The bursa is anteriorly open, supported by eight pairs of papillae (rays) arranged as follows <GP1, GP2, (GP3, CO), GP4, GP5d, GP6, (GP7d, GP8) phasmid>, where the distances between GP1-GP2, GP2-GP3 and GP6-GP7 are similar to each other, the distance between GP5d-GP6 is clearly larger and that between GP3-GP4 and GP4-GP5d is shorter; GP7d and GP8 are close to each other (Fig. 3).

In addition to its generic characters, *A. melissensis* n. sp. is characterized by its male tail characters described above. The new species described here is typologically identical to *A. rhodensis*, i.e., the male tail characters are shared by these two species (Kanzaki et al., 2017). These two species can be distinguished only by molecular phylogenetic status and mating experiments.

The species was originally isolated from a rotting starfruit (*Averrhoa carambola*) on September 21, 2009 on the Indian Ocean island of La Réunion in Saint Benoit (Melissa domain). The species epithet is after its type locality, Melissa domain.

Crosses between *A. melissensis* females and *A. rhodensis* or *A. freiburgensis* males (as well as the reciprocal crosses) did not result in progeny (Table 1), indicating that *A. melissensis* can be considered a distinct species from the two other known trioecious *Auanema* nematodes.

**Table 1:** Crosses were performed between *A. melissensis* and *A. rhodensis* or *A. freiburgensis*^.^ The number of crosses performed is denoted by “*n*.”

			Males	
		A. melissensis	A. rhodensis	A. freiburgensis
Females	*A. melissensis*		No offspring (*n* = 5)	No offspring (*n* = 11)
	*A. rhodensis*	No offspring (*n* = 5)		
	*A. freiburgensis*	No offspring (*n* = 8)		

**Figure 4:**
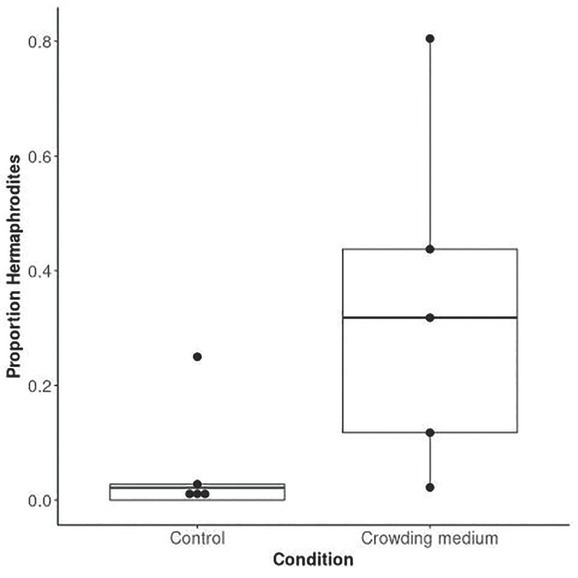
Hermaphrodite production is promoted when *A. melissensis* hermaphrodite mothers are cultured in the presence of a crowding cue. The boxplots were drawn from the proportion of hermaphrodites (number of hermaphrodites/number of hermaphrodites and females) under each condition for each replicate.

Similar to the other *Auanema* nematodes, all dauer larvae of *A. melissensis* developed into hermaphrodites (*n* = 48). To determine if the female *versus* hermaphrodite decision in *A. melissensis* is mediated by overcrowding cues as in *A. freiburgensis* (Zuco et al., 2018; Robles et al., 2021), we exposed hermaphrodite mothers to crowding cues. These cues were extracted from plates with high population densities of *A. melissensis*. Mothers exposed to the crowding cues produced a higher proportion of hermaphrodite progeny than under the control conditions (GLM *P*-value <0.01; Fig. 4, Table S5 in Supplementary Materials).

**Table S5:** Number of F1 males, females and hermaphrodites produced by selfing mothers in the absence (control) and presence (crowding) of a crowding cue.

Replicate	Male (%)	Female (%)	Hermaphrodite (%)	Total
Control 1	9 (11.7)	68 (88.3)	0 (0)	77
Control 2	3 (7.7)	35 (89.7)	1 (2.6)	39
Control 3	1 (7.7)	9 (69.2)	3 (23.1)	13
Control 4	4 (23.5)	13 (76.5)	0 (0.0)	17
Control 5	0 (0.0)	45 (97.8)	1 (2.2)	46
**Total Control**	**17 (8.9)**	**170 (88.5)**	**5 (2.6)**	**192**
Crowding 1	0 (0.0)	15 (88.2)	2 (11.8)	17
Crowding 2	0 (0.0)	15 (68.2)	7 (31.8)	22
Crowding 3	1 (2.2)	44 (95.7)	1 (2.2)	46
Crowding 4	0 (0.0)	27 (56.3)	21 (43.8)	48
Crowding 5	0 (0.0)	8 (19.5)	33 (80.5)	41
**Total Crowding**	**1 (0.6)**	**109 (62.6)**	**64 (36.8)**	**174**

For each replicate, we calculated the percentage of each sexual morph produced.

### Genome characteristics

The genome of *A. melissensis* was sequenced using a combination of pair-end and mate-pair Illumina short reads (see Section “Material and Methods”). The best assembly obtained was 59.7 Mb long and estimated to be 88.9% complete using the program BUSCO (version 5.2.2) with the Nematoda_odb10 database (Table 2). This BUSCO score was very close to the score obtained for *A. freiburgensis* (89.1%) and *A. rhodensis* (89.8%) (Table S3 in Supplementary Materials), which indicates that *A. melissensis*’ genome contains most genes. Statistics on the *A. melissensis* genome are summarized in Table 2. Repeat annotation estimated that 4.8 Mb (8%) of the genome was repetitive (Table S6 in Supplementary Materials).

**Table 2:** **Basic statistics on the genome of *A. melissensis* compared to that of *C. elegans***. **The nematoda_odb10 database was used for the BUSCO analysis**.

	A. melissensis PRJEB51845/ GCA_ 943334845.1	C. elegans PRJNA13758/ GCF_ 000002985.6
Number of scaffolds	7,511	6 + MT
Span (Mb)	59.7	100.3
GC content (%)	37.1	35.4
N50 (bp)	404,820 (*n* = 39)	17,493,829
Longest scaffold/ chromosome	2,171,611	20,924,180
*N* counts	5,769,006	0.00
Gaps	3,945	NA
Repeats	4,816,819 bp (8.07%)	(21.95%)
BUSCO (v5.2.2) score (on genome)	C: 88.9% [S: 88.5%, D: 0.4%], F: 1.4%, M: 9.7%, *n*: 3,131	C: 99.4% [S: 98.9%, D: 0.5%], F: 0.1%, M: 0.5%
No. of protein-coding genes	11,040	20,184
BUSCO (v5.2.2) score (on the proteome, using nematoda_ odb10, *n* = 3,131)	C: 89.7% [S: 77.2%, D: 12.5%], F: 1.1%, M: 9.2%	C: 100.0% [S: 74.8%, D: 25.2%], F: 0.0%, M: 0.0%

**Table S6:** Classification of the repeats by Repeat Masker.

Category	Number of elements*	Length occupied (bp)	Percentage of sequence
SINEs (all)	30	7,757	0.01
SINEs (ALUs)	0	0	0.00
SINEs (MIRs)	0	0	0.00
LINEs (all)	78	22,828	0.04
LINEs (LINE1)	0	0	0.00
LINEs (LINE2)	10	4,088	0.01
LINEs (L3/CR1)	14	5,507	0.01
LTR elements (all)	1,799	727,003	1.22
LTR elements (ERVL)	0	0	0.00
LTR elements (ERVL-MaLRs)	0	0	0.00
LTR elements (ERV_classI)	0	0	0.00
LTR elements (ERV_classII)	0	0	0.00
DNA elements	430	161,038	0.27
DNA elements (hAT-Charlie)	0	0	0.00
DNA elements (TcMar-Tigger)	1	807	0.00
Unclassified	6,850	2,109,448	3.53
Total interspersed repeats	NA	3,028,074	5.07
Small RNA	792	186,973	0.31
Satellites	167	46,755	0.08
Simple repeats	24,687	1,082,694	1.81
Low complexity	8,083	464,828	0.78

*Most repeats fragmented by insertions or deletions have been counted as one element. Masked sequences represented 4,816,819 bp (8.07%) of the genome of *A. melissensis* (59,698,091 bp of total length).

### Nigon analysis and putative X scaffolds

We used the BUSCO output from *A. melissensis* to look at the profile of Nigon elements across the scaffolds. Most scaffolds with BUSCO genes corresponded to only one Nigon element (190/224, or 84.8%). Of the scaffolds containing BUSCO genes of several Nigons, 26 (11.6%), 6 (2.6%), and 2 (0.8%) had a mix between 2, 3, and 4 Nigon elements, respectively. The data was provided as an Excel data file in the attachment.

Using the Nigon element concept, it was possible to identify putative scaffolds of the X chromosome. We considered putative X scaffolds containing at least three orthologs pertaining to the Nigon X element (Table 3).

**Table 3:** Putative X scaffolds. Scaffolds containing at least 3 Nigon X BUSCO genes were considered putative X scaffolds.

Scaffolds	Number of Nigon X BUSCO genes	Number of BUSCO genes of other Nigons
scaffold120 (CALQYR010007222.1)	10	0
scaffold167 (CALQYR010007273.1)	18	0
scaffold125 (CALQYR010007227.1)	5	0
scaffold146 (CALQYR010007250.1)	5	0
scaffold91 (CALQYR010007503.1)	12	0
scaffold79 (CALQYR010007489.1)	7	0
scaffold72 (CALQYR010007482.1)	6	0
scaffold42 (CALQYR010007449.1)	3	0
scaffold41 (CALQYR010007448.1)	3	0
scaffold190 (CALQYR010007299.1)	3	0
scaffold168 (CALQYR010007274.1)	3	0

### Phylogenetic position

The phylogenetic position of *A. melissensis* was determined relative to two other *Auanema* species, *A. rhodensis* (Tandonnet et al., 2019) and *A. freiburgensis* (Talal Al Yazeedi, pers. comm.), as well as *O. tipulae* (Gonzalez de la Rosa et al., 2021), *C. elegans* (WBcel235) and *P. pacificus* (el paco v4, GCA_000180635.4).

**Figure 5:**
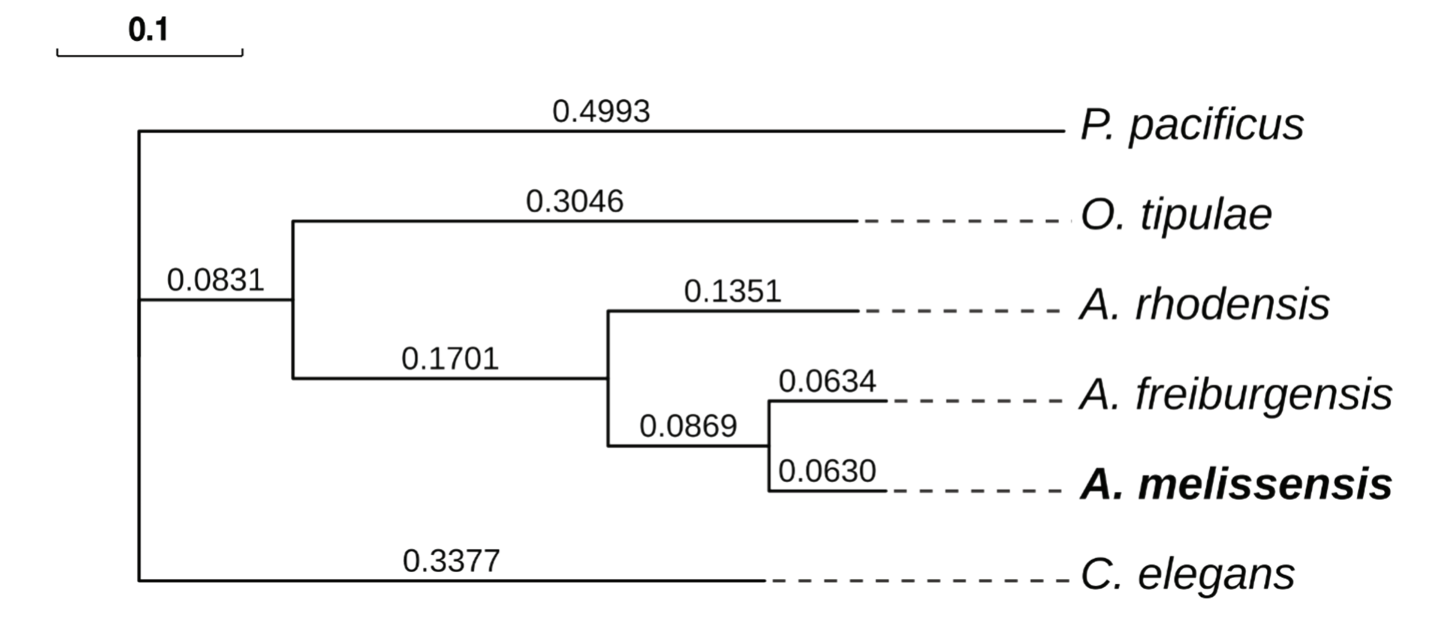
Phylogenetic position of *A. melissensis*. Single-copy BUSCO orthologs (2071) were used to build a concatenated alignment, which was subsequently used to construct the phylogenetic tree. The supermatrix alignment was 1,018,758 amino acids in length.

*Auanema melissensis* is placed as a sister taxon to *A. freiburgensis*, with *A. rhodensis* as an outgroup (Fig. 5). This phylogenetic position is the same as that previously reported, which was constructed based on rDNA and RNA polymerase II sequences (Kiontke et al., 2007; Kanzaki et al., 2017; Winter et al., 2017).

## Discussion

In this study, we report the genome and some biological characteristics of a non-described free-living nematode species, *A. melissensis*. This nematode shares many similarities with *A. rhodensis* and *A. freiburgensis*: it is trioecious, has a small genome (~60 Mb), and has fewer genes than *C. elegans* (11,040). Crosses between *A. melissensis* and *A. rhodensis* or *A. freiburgensis* did not result in viable progeny, indicating reproductive isolation.

The phylogenetic position based on the BUSCO single-copy orthologs places *A. melissensis* within *Auanema*, with *A. freiburgensis* as its closest relative. Nevertheless, *A. melissensis* has the same male tail morphological characteristics as *A. rhodensis*. This result indicates that *A. melissensis* can be regarded as a cryptic species of *A. rhodensis*, and can be distinguished from it with mating experiments and molecular sequences. Previous studies have also demonstrated other subtle differences in male sperm size (smaller) and sperm content (the presence of retained tubulin) in *A. melissensis* (aka *Rhabditis* sp. JU1783) compared to those of *A. rhodensis* and *A. freiburgensis* (Winter et al., 2017).

Similar to other *Auanema* species, the dauer larvae, a non-feeding developmental arrested stage usually linked to stress resistance and dispersal, obligatorily develops into a hermaphrodite adult (Félix, 2004; Chaudhuri et al., 2011a; Zuco et al., 2018; Robles et al., 2021). Similar to *A. freiburgensis*, crowding cues perceived by *A. melissensis* hermaphrodite mothers influence the sexual fate of their progeny (Zuco et al., 2018; Robles et al., 2021). In the absence of such cues, mothers produced primarily males and females. These outcrossing individuals can bring together new combinations of alleles that may increase fitness. The production of hermaphrodite-fated offspring, which obligatorily pass through the mobile and dispersive dauer stage, can colonize a new habitat without the need to find a mate. In *A. freiburgensis*, the molecular mechanisms controlling this maternal non-Mendelian inheritance involve the modulation of energy-sensing signaling activation of AMPK, downregulation of the insulin signaling (inhibition of *daf-18*), inhibition of the intracellular nutrient sensor mTORC1 complex and enhanced histone acetylation increase the proportion of hermaphrodite offspring (Robles et al., 2021). We hypothesize that similar mechanisms would be at play in *A. melissensis*.

In *A. freiburgensis*, it has been proposed that females and hermaphrodites may play different roles in the life cycle, with each sexual morph exhibiting adaptations specific to their part, stabilizing trioecy in the population (Adams et al., 2022). *A. freiburgensis* hermaphrodites invest resources in the expansion of the intestine, the major metabolic organ, which may enable them to meet the high energy cost of dispersal and reproduction (Adams et al., 2022). In contrast, the obligate outcrossing female diverts resources from intestinal development to invest in mate-finding behavior (Adams et al., 2022). Here we show that the sexual morphs of *A. melissensis* exhibit similar developmental differences. Unmated *A. melissensis* females are significantly shorter, with lesser developed intestines, than age-matched hermaphrodites, consistent with females limiting investment in growth and development. Further future analysis of sexual morph specialization in *A. melissensis* could help uncover how trioecy persists in the *Auanema* genus.

In this report, we also identified putative X chromosome scaffolds in the *A. melissensis* genome, using the concept of Nigon elements. Nigon elements are defined as groups of genes originally found on the seven ancestral chromosomes of Rhabditida (Tandonnet et al., 2019; Gonzalez de la Rosa et al., 2021). By finding *A. melissensis* orthologs to Nigon X genes it was possible to identify possible X chromosome scaffolds. This is particularly relevant in the *Auanema* species, as segregation and inheritance peculiarities concerning the X chromosome were observed in the related species *A. rhodensis*. In *A. rhodensis*, during the meiosis of XX hermaphrodites, the X homologs do not recombine either during oogenesis or spermatogenesis resulting in the production of nullo-X oocytes and 2X sperm (Tandonnet et al., 2018). This is in contrast with *A. rhodensis* XX females for which the X homologs pair and recombine during meiosis (Tandonnet et al., 2018). The X chromosome is also inherited from the father by the son in the event of a cross (female–male or hermaphrodite–male) due to the asymmetric partitioning of the cytoplasm during male spermatogenesis resulting in only X-bearing sperm being produced (Shakes et al., 2011; Tandonnet et al., 2018; Al-Yazeedi et al., 2022). To this date, the mechanisms controlling the unusual X chromosome segregation are unknown. Testing whether other trioecious members of the *Auanema* genus undergo similar processes and their possible consequences would be an interesting future avenue of research and the genomic X sequences are an important basis for such exploration.

## Abbreviations

GLM: generalized linear model
M9: M9 medium broth
NGM: nematode growth medium
WGS: whole genome sequencing
